# Low postoperative blood platelet count may be a risk factor for 3-year mortality in patients with acute type A aortic dissection

**DOI:** 10.1186/s13019-021-01623-7

**Published:** 2021-09-27

**Authors:** Guangyu Liu, Hongbai Wang, Qipeng Luo, Liang Cao, Lijing Yang, Cuntao Yu, Fuxia Yan, Su Yuan

**Affiliations:** 1 Anesthesia Center, Fuwai Hospital, National Center for Cardiovascular Diseases, Chinese Academy of Medical Sciences and Peking Union Medical College, No.167 North Lishi Road, Xicheng District, Beijing, 100037 China; 2 Pain Medicine Center, Peking University Third Hospital, 49 North Garden Road, Haidian District, Beijing, 100191 China; 3 Vascular Surgery Center, Fuwai Hospital, National Center for Cardiovascular Diseases, Chinese Academy of Medical Sciences and Peking Union Medical College, No.167 North Lishi Road, Xicheng District, Beijing, 100037 China

**Keywords:** Aortic diseases, Blood platelets, Vascular surgical procedures, Aortic replacement

## Abstract

**Background:**

Mortality and complications remain high after acute type A aortic dissection (ATAAD) open surgery and are associated with coagulation dysfunction. Platelets play an important role in the process of coagulation. This study explored the relationship between postoperative platelet counts and 3-year mortality after operation in patients with ATAAD undergoing open aortic repair surgery.

**Methods:**

Patients with ATAAD who underwent Total Arch Replacement and Frozen Elephant Trunk in Fuwai Hospital from 2011 to 2015 were selected for this study. The perioperative data were collected and sorted through the electronic clinical case system. Multivariate Logistic regression was used to analyze the risk factors for death within three years after surgery.

**Results:**

A total of 495 patients were included in the analysis. After correction for confounding factors, decreased postoperative platelet count remained an independent factor that was associated with lower mortality (OR = 0.918, 95% CI 0.853–0.988, *P* = 0.023).

**Conclusions:**

The study indicated that decreased postoperative platelet count may lead to increased 3-year mortality, in patients with ATAAD who underwent open aortic repair surgery.

**Supplementary Information:**

The online version contains supplementary material available at 10.1186/s13019-021-01623-7.

## Background

Aortic dissection is a relatively uncommon but potentially fatal condition. The incidence is approximately 2.6 to 3.5 per 100,000 population per year [1–4]. Surgery is the primary treatment for acute type A aortic dissection (ATAAD) [5]. However, the incidence of surgical complications, such as postoperative cerebral infarction and reoperation for bleeding, is high. Thus, perioperative mortality and long-term outcomes remain unsatisfactory [6–9].

One of the causes of poor prognosis is abnormal perioperative coagulation function. Heparin is necessary for the use of extracorporeal circulation, and it may cause postoperative hemorrhage. Protamine can antagonize the effect of heparin, but it may inhibit platelet function [10–12]. Surgical trauma, which is unavoidable, is also an essential factor in coagulation disorders. When an injury such as surgery causes bleeding, platelets play a crucial role in hemostasis. First, after the occurrence of trauma, platelets quickly adhere to the wound, then gather into a group, and form a hemostatic embolus. Second, platelets can activate, secrete, and interact with coagulation factors [13, 14].

It was hypothesized that, in patients with ATAAD after open aortic repair, a low platelet count may be associated with a poor prognosis. Therefore, we conducted a retrospective study to explore the relationship between postoperative platelet count and postoperative 3-year mortality in ATAAD.

## Methods

### Study population

The study was approved by the ethics committee of the research setting Hospital (Ethical approval number: 2017-877) and conducted under the guidance of the latest version of the Helsinki Declaration [15]. Written informed consent from all patients for this study was waived. The authors conducted this retrospective observational study of all consecutive patients with ATAAD who underwent total arch replacement and frozen elephant trunk (TAR + FET) between January 2011 and December 2015. ATAAD was defined by observing an intimal flap separating 2 lumina in the ascending aorta that occurred within 14 days of symptom onset [16]. The TAR + FET surgical technique has been described previously in detail and is viewed as a standard therapy for ATAAD requiring repair of the aortic arch [17]. The method of deep hypothermic circulatory arrest we adopted is stopping circulation at 18 degrees Celsius, not 25 degrees, which is the only difference between our study and Ma's article. The only difference is that this study adopts the method of deep and low temperature stopping circulation at 18 degrees Celsius. If the following techniques were used in the operation or the following conditions occurred, they were excluded from this study: hybrid procedure, off-pump surgery, or partial aortic arch replacement. Exclusion criteria were: patients with missing data from perioperative period and follow-up period. This article follows the STROBE Statement [18].

### Data acquisition

Demographic indicators that are were collected are: age, sex, body mass index (BMI), history of hypertension, history of smoking, history of alcohol consumption, history of diabetes, history of chronic obstructive pulmonary disease (COPD), history of peripheral vascular disease, and history of cerebrovascular disease. Preoperative blood pressure, preoperative cardiac ultrasonography, surgical history, and other preoperative conditions were also collected. Routine preoperative blood examinations, such as hemoglobin (Hb), white blood cell counts (WBCs), and platelet counts were also recorded. In addition, intraoperative indicators were collected, including lowest temperature during extracorporeal circulation, shutdown temperature of extracorporeal circulation, extracorporeal circulation duration and circulatory arrest duration. The data were collected and sorted through the electronic clinical case system of Fuwai Hospital.

Outcome indicators were collected by assessors blinded to the research design. The recorded outcome indicators included postoperative 3-year mortality, mortality in the hospital, length of postoperative hospital stay, and length of ICU stay. Other recorded outcomes included the rate of readmission to the ICU, cardiovascular dysfunction, neurological complications, respiratory complications, and digestive complications. Neurological complications included cerebral infarction, cerebral hemorrhage, hemiparalysis, and paraplegia. Respiratory complications included hydrothorax, pulmonary infection, respiratory insufficiency, and tracheal reintubation. Digestive system complications included postoperative liver insufficiency, gastrointestinal hemorrhage, intestinal obstruction, and acute pancreatitis. The diagnostic criteria of complications are listed in Additional file 1: table 1.

### Statistical analysis

Patients were divided into two groups for comparison based on the outcome of follow-up for death or survival. Continuous variables are presented as the mean ± standard deviation (SD), and categorical variables are reported as the counts (percentage). Time-to-event variables are presented as the median (95% confidence interval of median). Differences between groups were assessed using the chi-squared test or Fisher’s exact test for categorical variables, and independent samples Student’s t-tests or Mann–Whitney U test for continuous variables. Time-to-event variables were estimated with the Kaplan–Meier method, and differences between groups were compared with the log-rank test. Two-sided *P* values of less than 0.05 were considered statistically significant.

Logistic regression was used to analyze the risk factors for death within three years after surgery. Death within three years after surgery was taken as the dependent variable, and all preoperative, intraoperative, and postoperative indicators were put into single-factor regression analysis. Variables with a *P* value < 0.1 were put into multifactor regression equation. The backward stepwise method was adopted for the multifactor logistic regression equation, that is, all factors were included in the equation at the beginning. Then, multifactor regression is carried out step by step. During each step, if the *P* value of a factor was not less than 0.1, the factor was removed from the equation. Stata for Windows software version 16.0 was used for statistical analysis.

## Results

### Descriptive statistics

**Fig. 1:**
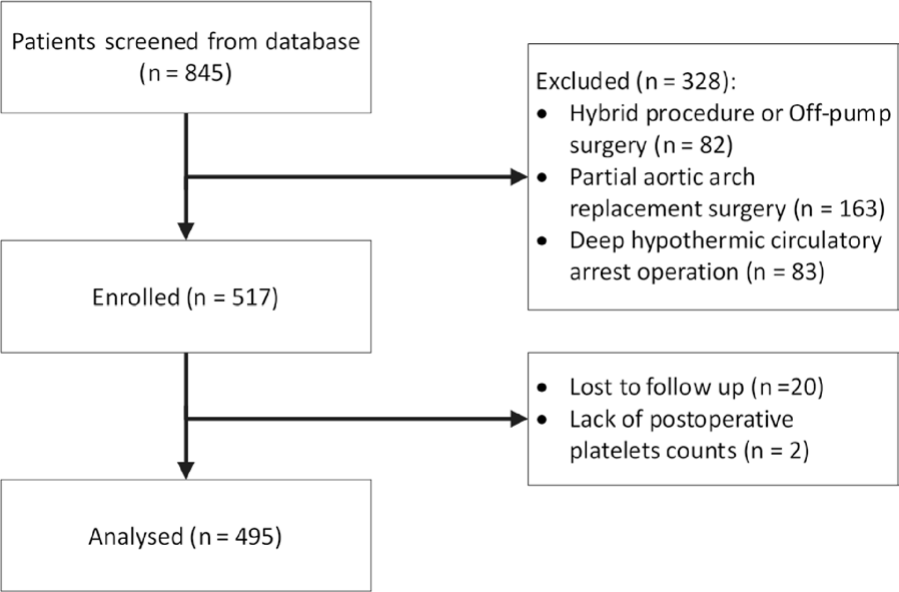
Research flowchart

All patients who underwent aortic dissection from 2011 to 2015 were screened. A total of 845 operation records were extracted from the database. Three hundred twenty-eight patients were excluded because the type of surgery did not meet the inclusion criteria. Twenty-two patients were excluded due to the lack of postoperative platelet count or loss to follow-up. Hence, a total of 495 patients were included in the analysis. The screening process and results are shown in Fig. 1.

Among all 495 patients, the mean age was 47.5 ± 10.7 years, and there were 110 females and 385 males. All patients were followed up for more than three years after surgery. In addition, the 3-year survival rate was 91.3%. In terms of preoperative indicators, there were no statistically significant differences between the two groups in BMI, time from onset to admission, preoperative complications, NYHA grading, preoperative ultrasound, preoperative WBC count, or HB count. However, there were statistical differences in sex, age, platelet count and other aspects between the two groups. The preoperative information of the patients is shown in Table 1.

**Table 1:** Preoperative and intraoperative variables of patients

	Overall (n = 495)	Survival (n = 452)	Death (n = 43)	*P* value
Gender, male	385 (77.78%)	357 (78.98%)	28 (65.12%)	0.037
Age, year	47.5 ± 10.7	47.1 ± 10.6	51.465 ± 10.119	0.011
BMI, kg/m^2^	25.57 ± 3.951	25.64 ± 4.026	24.86 ± 3.000	0.300
Time from onset to admission, h	3 [1, 10]	3 [1, 10]	2 [1, 7]	0.369
Hypertension	364 (73.54%)	335 (74.12%)	29 (67.44%)	0.343
Diabetes	10 (2.02%)	10 (2.21%)	0 (0%)	> 0.999
Peripheral vascular disease	9 (1.82%)	9 (1.99%)	0 (0%)	> 0.999
COPD	2 (0.40%)	2 (0.44%)	0 (0%)	> 0.999
Chronic kidney disease	24 (4.85%)	21 (4.65%)	3 (6.98%)	0.454*
Preoperative CNS disease	22 (4.44%)	21 (4.65%)	1 (2.33%)	0.710*
Hyperlipidemia	80 (16.16%)	76 (16.81%)	4 (9.3%)	0.201
The history of smoking	210 (42.42%)	192 (42.48%)	18 (41.86%)	0.938
The history of drinking	25 (5.05%)	23 (5.09%)	2 (4.65%)	> 0.999
History of cardiovascular surgery	35 (7.07%)	30 (6.64%)	5 (11.63%)	0.214*
Preoperative pain	459 (92.73%)	417 (92.26%)	42 (97.67%)	0.350*
NYHA grading:				
I	37 (7.47%)	33 (7.30%)	4 (9.3%)	0.437*
II	396 (80%)	364 (80.52%)	32 (74.42%)	
III	57 (11.52%)	51 (11.28%)	6 (13.95%)	
IV	5 (1.01%)	4 (0.88%)	1 (2.33%)	
MBP, mmHg	106 [96, 116.3]	106.7 [96.67, 116.7]	100.7 [93.33, 114]	0.091
LVEF, %	60 [58, 62]	60 [58, 62]	60 [59, 61]	0.8531
Left ventricular diameter, mm	51 [47, 56]	51 [47, 57]	49 [44, 55]	0.082
Pericardial effusion	69 (13.94%)	62 (13.72%)	7 (16.28%)	0.643
Preoperative WBC count, 10^9^/L	10.90 [8.220, 13.61]	10.83 [8.165, 13.58]	11.39 [8.490, 13.69]	0.448
Preoperative PLT count, 10^9^/L	169 [134, 220]	171 [137, 223]	147 [114, 176]	< 0.001
Preoperative Hb count, g/L	132 [116, 144]	132 [116, 144]	128 [117, 136]	0.165
Emergency operation	285 (57.58%)	258 (57.08%)	27 (62.79%)	0.469
Concurrent CABG	48 (9.7%)	42 (9.29%)	6 (13.95%)	0.289*
Concurrent Aortic root surgery	148 (29.9%)	135 (29.87%)	13 (30.23%)	0.960
Concurrent mitral valve plastic or replacement	8 (1.62%)	8 (1.77%)	0 (0.00%)	> 0.999
Blood loss, mL	900[600, 1200]	900 [600, 1200]	1000 [600, 1200]	0.311
Intraoperative red blood cell infusion, unit	0 [0, 0]	0 [0, 0]	0 [0, 2]	0.003
Intraoperative red blood cell infusion rate	56 (11.31%)	47 (10.40%)	9 (20.93%)	0.045
Frozen plasma infusion, mL	400 [0, 600]	400 [0, 600]	400 [0, 800]	0.093
Platelet infusion, unit	2 [1, 2]	2 [1, 2]	2 [2]	0.253
Lowest temperature in CPB	18.3 [17.7, 20.2]	18.3 [17.7, 20.25]	18.3 [17.7, 19.6]	0.719
Temperature when CPB is stopped	36.9 [36.6, 37.1]	36.9 [36.6, 37.1]	36.9 [36.6, 37.1]	0.514
Cardiopulmonary bypass time, min	186 [158, 217]	186 [155, 216]	194 [171, 261]	0.022
Clamping time, min	22 [19, 26]	22 [19, 25]	22 [20, 27]	0.273

Data are presented as mean ± SD, number (%), or median [interquartile range]

BMI, body mass index; COPD, chronic obstructive pulmonary disease; CNS, central nervous system; NYHA, New York Heart Association; MBP, mean blood pressure; LVEF, left ventricular ejection fraction; WBC, white blood cell; PLT, platelet; Hb, hemoglobin; CABG, coronary artery bypass grafting

^*^In these comparisons, Fisher's exact test was used

However, intraoperative indexes showed no statistically significant differences between the two groups in intraoperative combined operation type, blood loss, plasma and platelet infusion volume or clamping time. The *P* value of the two groups was less than 0.05 for the amount of red blood cell infusion and cardiopulmonary bypass (CPB) time, showing a statistical difference.

Among all enrolled patients, the in-hospital mortality rate was 8.28%. The median postoperative hospital stay was 12 days (IQR 9–16), and the median postoperative ICU stay was 48 h (IQR 33–88). There were 18 patients (3.64%) who underwent reoperation for hemorrhage after surgery, and 26 patients (5.25%) who underwent reoperation for other reasons. In terms of postoperative complications, there were 8 (1.62%) patients with cardiac dysfunction and 108 (21.82%) patients with central nervous system complications. The incidence of respiratory complications was 24.24%, and that of digestive complications was 33.74%. Postoperative data are shown in Table 2.

**Table 2:** Outcome results in patients

	Overall (n = 495)	Survival (n = 452)	Death (n = 43)	*P* value
In-hospital mortality	41 (8.28%)	0 (0.0%)	41 (95.35%)	< 0.001
Length of postoperative hospital, day	12 [9, 16]	12 [9, 16]	9 [4, 16]	0.001
Length of postoperative ICU stay, h	48 [33, 88]	48.5 [32, 88]	44 [35, 80]	0.5412
Reoperation for hemostasis	18 (3.64%)	13 (2.88%)	5 (11.63%)	0.003
Reoperation for other reasons	26 (5.25%)	18 (3.98%)	8 (18.60%)	< 0.001*
Re-admission to ICU	19 (3.84%)	13 (2.88%)	6 (13.95%)	0.003
Postoperative cardiac insufficiency	8 (1.62%)	3 (0.66%)	5 (11.63%)	< 0.001*
Postoperative complications of the CNS	108 (21.82%)	84 (18.58%)	24 (55.81%)	< 0.001
Postoperative complications of respiratory system	120 (24.24%)	93 (20.58%)	27 (62.79%)	< 0.001
Postoperative complications of digestive systems	167 (33.74%)	141 (31.19%)	26 (60.47%)	< 0.001

Data are presented as number (%), or median [interquartile range]

ICU, intensive care unit; CNS, central nervous system

^*^In these comparisons, Fisher's exact test was used

In the univariate logistic regression analysis, preoperative and intraoperative factors were analyzed, and factors with a *P* value < 0.1 were included in multivariate logistic regression. The results of univariate Logistic regression are shown in Additional file 1: table 2.

In the first step of multifactor regression, a total of 13 factors were put into the regression, including age, sex, CPB duration, red blood cell (RBC) infusion, fresh frozen plasma (FFP) infusion, postoperative platelet count, reoperation for hemostasis, reoperation for other reasons, readmission to the ICU, postoperative cardiac insufficiency, postoperative CNS complications, postoperative complications of the respiratory system, and postoperative infection. The backward-stepwise logistic regression method was used in multivariate regression, and in every step, factors whose *P* value was not less than 0.1 were removed from the regression model. After correction for confounding factors, postoperative platelet count remained an independent factor that was associated with lower mortality (OR = 0.918, 95% CI 0.853–0.988, *P* = 0.023) (Table 3). Other factors remaining in the logistic regression model were sex (OR = 3.213, *P* = 0.005), CPB duration (OR = 1.008, *P* = 0.012), readmission to the ICU (OR = 3.751, *P* = 0.041), postoperative cardiac insufficiency (OR = 13.614, *P* = 0.006), postoperative CNS complications (OR = 2.986, *P* = 0.004) and postoperative complications of the respiratory system (OR = 3.976, *P* < 0.001).

**Table 3:** Factors in association with postoperative death in follow up

	Univariate analyses^a^		Multivariate analysis^b^	
	OR (95% CI)	*P* value	OR (95% CI)	*P* value
Age, year	1.040 (1.009, 1.072)	0.012	–	–
Gender, male	2.013(1.034, 3.921)	0.040	3.213 (1.426, 7.240)	0.005
CPB duration, min	1.009 (1.003, 1.014)	0.002	1.008 (1.002, 1.015)	0.012
Red blood cells infusion, U	1.200 (1.066, 1.351)	0.003	–	–
Fresh frozen plasma infusion, ml	1.001 (1.000, 1.001)	0.007	–	–
Postoperative platelets count, 10*10^9^/L	0.897 (0.837, 0.961)	0.002	0.918(0.853, 0.988)	0.023
Reoperation for hemostasis	4.443 (1.504, 13.128)	0.007	–	–
Reoperation for other reasons*	5.511 (2.238, 13.572)	< 0.001	–	–
Readmission to ICU	5.476 (1.967, 15.245)	0.001	3.751 (1.055, 13.332)	0.041
Postoperative cardiac insufficiency	19.693 (4.532, 85.572)	0.001	13.614 (2.124, 87.248)	0.006
Postoperative complications of the CNS	5.534 (2.898, 10.567)	< 0.001	2.986 (1.411, 6.319)	0.004
Postoperative complications of the respiratory system	6.514 (3.370, 12.592)	< 0.001	3.976 (1.860, 8.498)	< 0.001
Postoperative infection	2.206 (1.073, 3.826)	0.030	–	–

CPB, Cardiopulmonary bypass, ICU, Intensive care unit, CNS, Central nervous system

^*^Other postoperative operations other than postoperative hemostasis

^a^Postoperative death was modeled as a function of a single factor in the univariate logistic regression analyses

^b^Postoperative death was modeled as a function of several factors with a *P* value < 0.10 in the univariate analyses. Multivariate Logistic regression analysis was performed using a Backward stepwise procedure. Hosmer–Lemeshow test of goodness of fit of the model: χ^2^ = 1.98, df = 6, *P* = 0.922

## Discussion

The results of this study showed that in type A aortic dissection patients who underwent open aortic repair surgery, a low postoperative platelet count may be associated with increased 3-year mortality.

Although the complication rate and mortality have declined as techniques have improved, patient outcomes remain to be improved. Hemostasis is a vital process in wound healing, and affects the length of hospitalization. In this process, platelets play an important role. One aspect is that during surgery and CPB, large amounts of platelets are consumed, resulting in a platelet count decrease. Another aspect that cannot be ignored is that, after acute aortic dissection, a large number of clots will form in the dissection, which will also consume a large number of platelets in human body. When the platelet count is insufficient, postoperative wound hemostasis may be attenuated, resulting in poor prognosis.

Several studies have explored the association of platelets with postoperative outcomes after cardiac surgery. Ranucci [19] conducted PLATFORM study, which was a prospective cohort study, involving 490 cardiac surgery patients who underwent extracorporeal circulation, and found that platelet function was significantly associated with the risk of massive hemorrhage after cardiac surgery. Some studies have also found that abnormal platelet function is associated with poor prognosis in patients after cardiac surgery [20]. In addition, when fibrinogen or platelets are transfused into patients with progressive hemorrhage after cardiac surgery, they can significantly improve the functions of blood coagulation and platelets, thereby reducing the amount of blood loss [21]. A meta-analysis of 1720 patients who underwent coronary bypass surgery found that postoperative platelet transfusion was associated with poor prognosis, but platelet transfusion might be a surrogate marker of sicker patients to some extent, rather than the direct cause of adverse factors [22].

Several previous studies have explored the relationship between platelets and prognosis in patients who underwent aortic dissection. Chen et al. [23] found that in patients with acute type A aortic dissection, platelet count, lymphocyte to neutrophil ratio and lymphocyte to monocyte ratio may predict postoperative death of patients. Although the multifactor prediction model was not of statistical significance, he believed that the combined use of the three in multifactor analysis was necessary. While Liu et al. [24] in a retrospective study, found WBC, neutrophil and platelet counts might the better predictors of in hospital mortality than platelet to lymphocyte ratio and neutrophil to lymphocyte ratio. Sbarouni et al. [25] observed 120 consecutive patients with ATAAD, and found that the ratio of platelets to lymphocytes was associated with mortality, and the best critical value was 159, which showed a sensitivity of 53% and a specificity of 86%. These two studies suggest that platelet counts may be associated with prognosis, but not be an independent risk factor. For one thing, the study revealed a possible relationship between postoperative platelet levels and mortality at 3 years in patients with ATAAD. In addition, unlike most studies, where the platelet count admitted to hospital is a patient's baseline value, the postoperative platelet count in our study is the result of treatment by the anesthesiologist and surgeon. Further study of this project could give us a postoperative target value to guide perioperative platelets transfusion in patients with ATAAD.

There are some limitations in present study. Platelet counts instead of platelet function tests were used. There are several methods to measure platelet function, such as light transmission platelet aggregation, lumiaggregometry and impedance platelet aggregation [26–28]. Currently, there is no widely used laboratory test method to measure platelet function [29]. The platelet count used in this study is more widely used in clinical practice, making the results of the study more generally applicable. On the other hand, the research was a retrospective study. Prospective studies are needed to verify the findings.

## Conclusions

This study indicated that decreased postoperative platelet count may lead to increased 3-year mortality, in patients with ATAAD who undergo open aortic repair surgery. It is necessary to infuse ATAAD patients with platelets in this type of surgery to maintain a high platelet count.

## Supplementary Information

**Additional file 1: Table 1.** Diagnostic criteria for each postoperative complications. **Table 2.** The results of univariate Logistic regression.

## Data Availability

Due to the policy of management, raw data and material are not available. Reasonable requests for access to the datasets used and/or analyzed during this study can be made to the corresponding author.

## Abbreviations

ATAAD: Acute Type A Aortic Dissection
TAR + FET: Total Arch Replacement and Frozen Elephant Trunk
BMI: Body mass index
COPD: Chronic obstructive pulmonary disease
WBC: White blood cell
HB: Hemoglobin
ICU: Intensive care unit
CPB: Cardiopulmonary bypass
RBC: Red blood cell
FFP: Fresh frozen plasma
CNS: Central nervous system
