# Supplementary material for: Low postoperative blood platelet count may be a risk factor for 3-year mortality in patients with acute type A aortic dissection
Source: J Cardiothorac Surg. 2021 Sep 27;16:274. doi: 10.1186/s13019-021-01623-7 (PMC8477470; doi:10.1186/s13019-021-01623-7)
Supplement: Supplementary file 1 — Additional file 1: Table 1. Diagnostic criteria for each postoperative complications. Table 2. The results of univariate Logistic regression. [file 13019_2021_1623_MOESM1_ESM.docx]

Table 1. Diagnostic criteria for each postoperative complication.

| Complications | Requirements for acceptance |
| --- | --- |
| Circulatory dysfunction | Requirement of vasoconstrictors or inotropic agents for more than 24 hours after surgery. |
| Arrhythmia | New onset changes from the normal sequence of electrical impulses. Confirmed by 12‐lead electrocardiogram and necessitated medical treatment and/or cardioversion. |
| Cerebral infarction, cerebral hemorrhage, hemiparalysis, or paraplegia | Newly emerging persistent symptoms and confirmed by corresponding neuroimaging. |
| Hydrothorax | Continuous drainage of pleural fluid and prolonged placement of the drainage tube. |
| Pulmonary infection | New infiltrate was shown in Lung images, leukocytosis and body temperature over 38°C. |
| Respiratory insufficiency | PaO_2_/Fi0_2_ < 200 or prolonged mechanical ventilation time |
| Acute renal failure | New onset renal failure that required renal replacement therapy. |
| Liver insufficiency | hyperbilirubinemia, clinically apparent ascites, prolonged coagulopathy requiring frozen fresh plasma, and/or hepatic encephalopathy. |
| Gastrointestinal bleeding | Postoperative hematemesis, hematochezia and/or occult blood in stool are positive. |
| Intestinal obstruction | Symptoms such as abdominal pain, vomiting, abdominal distension, and cessation of exhaust and defecation are confirmed by abdominal X-ray. |
| Acute pancreatitis | Abdominal pain, nausea, vomiting and other related symptoms, and confirmed by blood and urine test results. |

PT= prothrombin time; PTA= prothrombin activity; INR = international normalized ratio; APTT = activated partial thromboplastin time.

Table 2. The results of univariate Logistic regression.

| Variables | Results of Univariate Logistic regression | | |
| --- | --- | --- | --- |
|  | N | OR | *P* value |
| Age, year | 495 | 1.04 | 0.012 |
| Gender, male | 495 | 2.013 | 0.040 |
| BMI, kg/m^2^ | 495 | 0.948 | 0.213 |
| MBP, mmHg | 495 | 0.987 | 0.186 |
| Hypertension | 495 | 0.723 | 0.345 |
| Diabetes | 495 | 1 | - |
| Peripheral vascular disease | 495 | 1 | - |
| The history of smoking | 495 | 0.975 | 0.938 |
| Hyperlipidemia | 495 | 0.507 | 0.209 |
| The history of drinking | 495 | 0.910 | 0.900 |
| Preoperative pain | 495 | 3.525 | 0.220 |
| Chronic kidney disease | 495 | 1.539 | 0.500 |
| Preoperative CNS disease | 495 | 0.489 | 0.490 |
| NYHA grading | 495 | 1.165 | 0.640 |
| History of cardiovascular surgery | 495 | 1.851 | 0.229 |
| Preoperative cardiac sonography |  |  |  |
| Pericardial effusion | 495 | 1.223 | 0.643 |
| LVEF, % | 495 | 1.028 | 0.422 |
| Left ventricular diameter, mm | 495 | 0.967 | 0.127 |
| Aortic ring diameter, mm | 446 | 0.984 | 0.255 |
| Diameter of aortic sinus, mm | 459 | 0.993 | 0.371 |
| Diameter of ascending aorta | 486 | 0.985 | 0.138 |
| Preoperative laboratory results |  |  |  |
| WBC count, 10^9^/L | 495 | 1.029 | 0.424 |
| PLT count, 10^9^/L | 494 | 0.988 | 0.001 |
| Hb count, g/L | 495 | 0.991 | 0.250 |
| pH in blood gas analysis | 480 | 0.997 | 0.958 |
| PaCO_2_ in blood gas analysis, mmHg | 441 | 1.000 | 0.969 |
| Intraoperative |  |  |  |
| Bloodloss, ml | 495 | 1.000 | 0.201 |
| RBC infusion, U | 495 | 1.200 | 0.003 |
| Fresh frozen plasma infusion, ml | 495 | 1.001 | 0.007 |
| Platelet infusion, U | 495 | 1.071 | 0.617 |
| CPB duration, min | 495 | 1.009 | 0.002 |
| Lowest temperature, ℃ | 495 | 0.929 | 0.339 |
| Clamping duration, min | 495 | 1.018 | 0.360 |
| Deep hypothermic circulatory arrest time, min | 495 | 0.800 | 0.520 |
| Emergency operation | 495 | 1.269 | 0.470 |
| Concurrent CABG | 495 | 1.018 | 0.960 |
| Concurrent aortic root surgery | 495 | 1.018 | 0.354 |
| Concurrent mitral valve surgery | 487 | 1.583 | 0.327 |
| Postoperative hemostasis | 495 | 5.511 | <0.001 |
| Other reoperations after the operation | 495 | 4.443 | 0.007 |
| Postoperative respiratory complications | 495 | 2.401 | 0.273 |
| Postoperative digestion complications | 495 | 3.373 | <0.001 |
| Postoperative acute kidney injury | 495 | 2.129 | 0.495 |
| Postoperative complications of the central nervous system | 495 | 5.534 | <0.001 |
| Postoperative infection | 495 | 2.026 | 0.030 |
| Postoperative cardiac insufficiency | 495 | 19.693 | <0.001 |
| Other postoperative complications | 495 | 1.052 | 0.962 |
| Postoperative ICU stay, h | 495 | 1.000 | 0.960 |
| Postoperative ICU readmission | 495 | 5.476 | 0.001 |
